# Research of cervical microbiota alterations with human papillomavirus infection status and women age in Sanmenxia area of China

**DOI:** 10.3389/fmicb.2022.1004664

**Published:** 2022-10-06

**Authors:** Jintao Hu, Yuhan Wu, Lili Quan, Wenjuan Yang, Jidong Lang, Geng Tian, Bo Meng

**Affiliations:** ^1^Faculty of Engineering and Information Technology, The University of Melbourne, Parkville, VIC, Australia; ^2^Genesis (Beijing) Co., Ltd., Beijing, China; ^3^College of Life Sciences, University of Chinese Academy of Sciences, Beijing, China; ^4^Department of Gynecology, Sanmenxia Central Hospital of Henan University of Science and Technology, Sanmenxia, Henan, China

**Keywords:** cervical cancer, TCT, HPV - human papillomavirus, microbiota, diversity

## Abstract

**Background:**

Human papillomavirus (HPV) infection is the leading cause of cervical cancer. More and more studies discovered that cervical microbiota (CM) composition correlated with HPV infection and the development of cervical cancer. However, more studies need to be implemented to clarify the complex interaction between microbiota and the mechanism of disease development, especially in a specific area of China.

**Materials and methods:**

In this study, 16S rDNA sequencing was applied on 276 Thin-prep Cytologic Test (TCT) samples of patients from the Sanmenxia area. Systematical analysis of the microbiota structure, diversity, group, and functional differences between different HPV infection groups and age groups, and co-occurrence relationships of the microbiota was carried out.

**Results:**

The major microbiota compositions of all patients include *Lactobacillus iners*, *Escherichia coli*, *Enterococcus faecalis*, and *Atopobium vaginae* at species level, and *Staphylococcus*, *Lactobacillus*, *Gardnerella*, *Bosea*, *Streptococcus*, and *Sneathia* in genus level. Microbiota diversity was found significantly different between HPV-positive (Chao1 index: 98.8869, *p* < 0.01), unique-268 infected (infections with one of the HPV genotype 52, 56, or 58, 107.3885, *p* < 0.01), multi-268 infected (infections with two or more of HPV genotype 52, 56, and 58, 97.5337, *p* = 0.1012), other1 (94.9619, *p* < 0.05) groups and HPV-negative group (83.5299). Women older than 60 years old have higher microbiota diversity (108.8851, *p* < 0.01, *n* = 255) than younger women (87.0171, *n* = 21). The abundance of *Gardnerella* and *Atopobium vaginae* was significantly higher in the HPV-positive group than in the HPV-negative group, while *Burkholderiaceae* and *Mycoplasma* were more abundant in the unique-268 group compared to the negative group. *Gamma-proteobacteria* and *Pseudomonas* were found more abundant in older than 60 patients than younger groups. Kyoto Encyclopedia of Genes and Genomes (KEGG) and Clusters of Orthologous Groups (COG) analysis revealed the effects on metabolism by microbiota that the metabolism of cells, proteins, and genetic information-related pathways significantly differed between HPV-negative and positive groups. In contrast, lipid metabolism, signal transduction, and cell cycle metabolism pathway significantly differed between multi-268 and negative groups.

**Conclusion:**

The HPV infection status and age of women were related to CM’s diversity and function pathways. The complex CM co-occurrent relationships and their mechanism in disease development need to be further investigated.

## Introduction

Cervical cancer is one of the most common malignant tumours among women. Clinical and epidemiological studies have determined that persistent Human Papillomavirus (HPV) infection is the leading risk factor for developing cervical cancer (Khan et al., 2020). The average time interval from carcinogenic HPV infection to cervical cancer progression is 25–30 years. The Thin-prep Cytologic Test (TCT) and HPV DNA were recommended to be used for HPV infection and cervical cancer status determination (Zhang et al., 2020). TCT detects the morphology of the cells and analyses the bacterial population in the sample through 16S rDNA (Deoxyribonucleic Acid) (Liu et al., 2020), which makes it possible to perform large-scale testing of samples.

Recent research revealed that microbiota might be a significant factor in the relationship between HPV and cervical cancer. Klein et al. found that changes in the cervical microbiota (CM) are related to cervical cancer (Kunene and Mahlangu, 2017). Some studies have shown that cervical/vaginal *Lactobacilli* can produce lactic acid that inhibits the growth of bacteria associated with bacterial vaginosis (BV) and viral infections (Polatti, 2012). The change in the proportion of microorganisms is related to pathological changes in the reproductive tract. In addition, recent studies have shown a clear correlation between microbiota and HPV infection (Libby et al., 2008; Anahtar et al., 2015). A report also explained a positive correlation between cervical HPV infection and BV-related microbiota (Gillet et al., 2011; Lee et al., 2013). Therefore, microbiota may play an important role in between, which implies that the reveal of the mechanism microbiota play is beneficial to comprehend the HPV infection and cancer evolution. However, the current research results have not clarified the mutual influence (Libby et al., 2008; Anahtar et al., 2015). So far, there are relatively few studies on the association amongst CM, cervical cancer and HPV infection, especially in China, which prompted this research to be conducted.

Therefore, samples from 276 patients were obtained to conduct microbiota research for further analysis. This research aimed to explore the relationship between the HPV infection and CM changes by analysing microbiota changes and the HPV infection concerning HPV infection group, different genotype HPV groups, and the impact of microbiota on cell and metabolic functions, as well as to explore the microbiota changes amongst the group divided by ages in a cohort of populations in Sanmenxia, Henan Province. Aside from that, this research also provides a new reference basis for further understanding the CM’s overall characteristics.

## Materials and methods

### Study population and specimen collection

A total of 276 cervical lesion samples were collected from patients at Sanmenxia Central Hospital for high-grade squamous intraepithelial lesion (HSIL) screening. The hospital’s medical ethics committee approved this study, and all experiments were carried out following the relevant guidelines and regulations. A fluorescent HPV genotyping kit (Bioperfectus Technologies, Jiangsu, China) was used to analyse the samples for confirmation and subsequent HPV typing. Women who came to Sanmenxia central hospital to do cervical tests in 2019, including TCT test and HPV genotype test, were enrolled in this study. Those samples were not qualified for further study, or low-quality sequencing results were excluded from the study.

### DNA extraction

After Pap Smear preparation, 1-ml of the remaining fluid sample was used for DNA isolation. According to the manufacturer’s instructions, the total Genomic DNA sample was extracted using TIANamp Micro DNA Kit (TIANGEN, Beijing, China). The double-stranded (ds) DNA was quantified using a Nanodrop 2000 and Qubit dsDNA HS assay kit (Thermo Fisher Scientific, Inc., Waltham, MA, USA). The average fragment size of DNA (> 5 Kbp) was measured (identified by comparison to a DL2000 PLUS DNA Ladder, Life Technologies, Carlsbad, CA, USA) on a 1.0% agarose gel in 1×TAE buffer on a Bio-Rad CHEF DRII system.

### Sequencing and bioinformatic processing

To build a sequencing library, use PCR primers to amplify the V3–V4 hypervariable region of the 16S rDNA gene. This area provides sufficient information for the taxonomic classification of microbial communities in specimens related to human microbiota research and is used by the Human Microbiota Project.

Then use Agencourt AMPure XP (Beckman Coulter, Indianapolis, Indiana) to select the product size in a ratio of 0.9 and group in equal moles. Then, a Qubit 2.0 fluorometer (Life Technologies) was used to quantify the pool of the selected size and loaded into the Illumina HiSeq flow cell (Illumina, Inc., San Diego, CA, USA) 2 × 250. Mix the library with the Illumina-generated PhiX control library and our genomic library, and use fresh NaOH for denaturation. Perform image analysis, base calls, and data quality assessment on the MiSeq instrument.

### Data analysis

Paired-end sequencing (2 × 250) was performed on Illumina HiSeq. The FASTQ conversion of the original data file is completed after demultiplexing with MiSeq Reporter. The quality assessment of FASTQ files is carried out using FASTQC,^1^ then quality filtering is performed using the FASTX toolkit.^2^ The high-quality reads used for analysis (where 80% of the base Q scores > 20 reads) and reads with unknown bases (“N”) are discarded. The remaining steps are performed using the Quantitative Analysis of Microbial Ecology (QIIME) software package version 1.8. Use UCHIME to filter chimeric sequences and use UCLUST to group sequences into the Operational Taxa Unit (OTU) with a similarity threshold of 97%. The Ribosomal Database Program (RDP) classifier trained using Greengenes 16S rDNA database (v13.8) assigns all OTUs to all OTUs with a confidence threshold of 80%. OTUs with an average abundance of less than 0.005% are eliminated. Use PyNAST v1.2 for multiple sequence alignment and FastTree v2.1 to construct a phylogenetic tree. The alpha and beta diversity indicators are calculated according to the method implemented in QIIME. A Phylogenetic Investigation of Communities by Reconstruction of Unobserved States (PICRUSt) is used to predict the orthologs from the Kyoto Encyclopedia of Genes and Genomes (KEGG) and Clusters of Orthologous Groups (COG) (i.e., the count of functional genes) for each sample and inferred genes. The count is allocated to the KEGG and COG channels.

### Statistic analysis

The numeric values representing the relative abundance of the OTU were analysed for statistical significance by performing a *t*-test. The statistical software in Sigma Plot version 11.0 was used (Systat Software Inc., San Jose, CA, USA). A total of 95% confidence intervals were estimated for sensitivity and specificity with a binomial test. Differences in means with *p*-values less than 0.05 were considered statistically significant. When tests for normality failed, the non-parametric data were analysed using the Mann–Whitney Rank Sum test and median values were determined.

## Results

### Patient cohort sociodemographic and characteristics

Complete data were available for 276 cervical smear samples taken from eligible women. The patients’ characteristics of the study population were summarised in Table 1, and detailed information about each patient was shown in Supplementary Table 1. The mean age of the patient’s cohort was 44.68 ± 10.94, from 17 to 80 years old. There were 83 HPV-infected patients in the 255 candidates under 60 years old and 11 HPV-infected patients in the 21 candidates over 60. The infection status of the subjects, including HPV single-type infection and multi-type infection. Five majors HPV types were detected, including HPV16 (*n* = 10), HPV39 (*n* = 7), HPV52 (*n* = 8), HPV56 (*n* = 7), and HPV58 (*n* = 12), and 36 subjects were infected by other HPV genotypes. Several cases were detected as unique-268 infections (52, 56, or 58 genotype infections, *n* = 27) among the 80 single infections and other related co-infections were multi-268 infections (52, 56, and 58 genotypes infecting two or more, *n* = 13) among the 14 multiple infections. Hence, the number of infections excluding 52, 56, and 58 were 54 infections, denoting as other1. Among the 21 subjects diagnosed with Cervical Intraepithelial Neoplasms (CIN), thirteen patients were infected by HPV and eight were not. While 81 HPV-positive and 174 (255 totally) HPV-negative subjects were identified in subjects without CIN status.

**TABLE 1:** Patients age and infection status.

Characteristics	Positive	Negative
**Age**		
*A*	83	172
A60	11	10
**HPV infection situation**		
Single-type infection	80	–
HPV16	10	–
HPV39	7	–
HPV52	8	–
HPV56	7	–
HPV58	12	–
Other	36	–
Unique-268	27	–
Multi-type infection	14	–
Muiti-268	13	–
Other1	54	–
**CIN suffering**		
CIN	13	8
Normal	81	174

*A* refers to age under 60, A60 refers to age over 60; Unique-268 refers to HPV single infection with one of 52, 56, or 58 subtypes, multi-268 refers to HPV multiple infections with at least two of 52, 56, or 58 subtypes, other1 refers to HPV infections besides 52, 56, and 58 subtypes. *Denotes no data.

### Baseline composition similar but the structure of the cervical microbiota variant between samples

The average length of the PCR product was about 465 bps from V3 to V4 segments of the 16S rDNA genes. After sequencing, the data amount and quality of each sample were evaluated by GC content (averagely 52.8%), Q20 value (averagely 96.2%), Q30 value (averagely 92%), and effectiveness (averagely 73.3). The detailed information of each sample was summarised in Supplementary Table 1. After removing the low-quality sequencing reads, a total number of 14,435,817 clean tags and an average of 79,095 tags of each specimen (each specimen generating at least 8,807 clean tags) were obtained. After the removal of singletons and rare OTUs (species abundance less than 0.005%), a total of 11 phyla, 17 classes, 30 orders, 53 families, 99 genera, and 132 species (Supplementary Table 2) were identified from the study cohort sequencing data.

The abundance of the ten most abundant bacteria families was summarised and listed in Supplementary Table 3. *Lactobacillaceae*, *Enterobacteriaceae*, *Staphylococcaceae*, *Enterococcaceae*, *Bifidobacteriaceae*, *Beijerinckiaceae*, *Streptococcaceae*, *Leptotrichiaceae*, *Burkholderiaceae*, and *Corynebacteriaceae* were the top 10 most abundant bacteria families in all the samples. The corresponding distribution figure is shown in Supplementary Figure 1. Results showed that the microbiota structure variant obviously between samples, but there were still pattern similarities between parts of the samples. As previously published papers mentioned, the CM was classified into five clusters (Zhou et al., 2020). Our results showed sample clusters with highly abundant *Escherichia-Shigella*, *Lactobacillus*, *Enterococcus*, *Staphylococcus*, and *Lactobacillus* in each cluster, respectively (Supplementary Figure 1). Moreover, some samples showed different characteristics with more bacterium types in the structure. Sample alpha diversity, including Chao1, ACE, Shannon, and Simpson indexes of all the samples, was calculated and summarised in Supplementary Table 4, indicating that the diverse samples differentiated significantly between samples.

### Differences in human papillomavirus infection status or age are highly relevant to the change of microbiota structure and diversity

**FIGURE 1:**
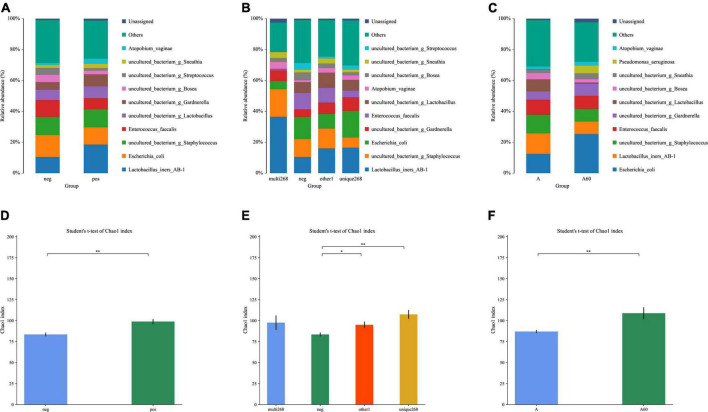
Relative abundance and alpha-diversity analysis of this study. **(A)** Microbiota relative abundance distribution of HPV-positive and HPV-negative patient groups; **(B)** microbiota relative abundance distribution of HPV-positive unique-268 infection and multiple 268 infection, and HPV-negative patient groups; **(C)** microbiota relative abundance analysis of the elder (age > 60) and the younger (age ≤ 60) patient groups; alpha-diversity analysis; a-diversity comparison bar diagram, **(D)** Chao1 index for normal (without CIN) HPV-positive and HPV-negative patient groups, **(E)** Chao1 index for HPV-positive unique-268, and multiple 268 infections, HPV-negative patient groups, **(F)** Chao1 index for the elder (age > 60) and the younger (age ≤ 60) patient groups. neg: HPV-negative without CIN, pos: HPV-positive without CIN, multi-268: HPV 52, 56, and 58 genotypes multiple infection (regardless of CIN), unique268: HPV 52, 56, or 58 genotypes unique (single) infection (regardless of CIN), other1: other HPV infection situations (regardless of CIN). A: age under 60, A60: age above 60, *p-value < 0.05, ^**^p-value < 0.01.

This study explored the difference in microbiota composition and diversity between sample groups. Two group pairs showed significant differences in HPV infection status and age. The relative abundance results of different HPV infection status groups (Figure 1A and Supplementary Table 4) showed the following conclusions: (1) Both normal sample groups with HPV infection (*n* = 78) and non-infection (*n* = 187) were predominated by the following types of bacteria, including *Lactobacillus iners* (HPV-positive/HPV-negative: 0.185/0.104), *Escherichia coli* (0.112/0.143), *Enterococcus faecalis* (0.071/0.108), and *Atopobium vaginae* (0.033/0.013) in species level, and *Staphylococcus* (0.116/0.117), *Lactobacillus* (excluding *Lactobacillus iners AB1*, 0.078/0.069), *Gardnerella* (0.076/0.048), *Bosea* (0.026/0.049), *Streptococcus* (0.015/0.043), and *Sneathia* (0.031/0.020) in genus level; (2) In the microbiota structure (relative abundance), which may illustrate a structure change before and after the HPV infection (Figure 1A and Supplementary Table 4). Further study on the unique-268 (sum of 52, 56, and 58 genotype HPV infection cases, *n* = 27) and multi-268 (co-infections with one or two HPV 52, 56, and 58 genotypes, *n* = 13) types of HPV infected samples, the microbiota detected was almost the same as the HPV-positive except for the composition proportion (Figure 1B and Supplementary Table 4). Additionally, the age group under 60 (*n* = 255) and above 60 (*n* = 31) detected a similar microbiota with a different proportion of composition (Figure 1C and Supplementary Table 4).

Alpha diversity was applied to analyse the complexity of species diversity. Regarding the analysis, the HPV-positive group had higher diversity (Chao1 index: 98.8869, *p* < 0.01) compared to the negative group (Chao1 index: 83.5299, Figures 1C,D and Supplementary Table 5). This disparity in microbiota diversity resulted from the significant differences between the two cases and evidenced structural change. Unique-268 (Chao1 index: 107.3885, *p* < 0.01), multi-268 (Chao1 index: 97.53) and other1 (Chao1 index: 94.9619, *p* < 0.05) had a higher microbiota diversity compared to the HPV-negative groups (Figure 1E and Supplementary Table 5). In addition, compared to younger patients, the elder group (age > 60, *n* = 31) has a higher diversity with statistical significance (Chao1 index: 108.8851, *p* < 0.01) than the younger group (age ≤ 60, *n* = 255, Chao1 index: 87.0171, Figure 1F and Supplementary Table 5), which also demonstrates the difference in microbiota structure. Hence, this diversity analysis indicates the following conclusions: (1) The normal HPV-positive groups and (2) unique-268 HPV and other1 infections were more diverse in microbiota than the HPV-negative groups, while (3) the age group over 60 had higher diversity concerning the microbiota.

### Bacteria biomarkers were identified in different subject groups

**FIGURE 2:**
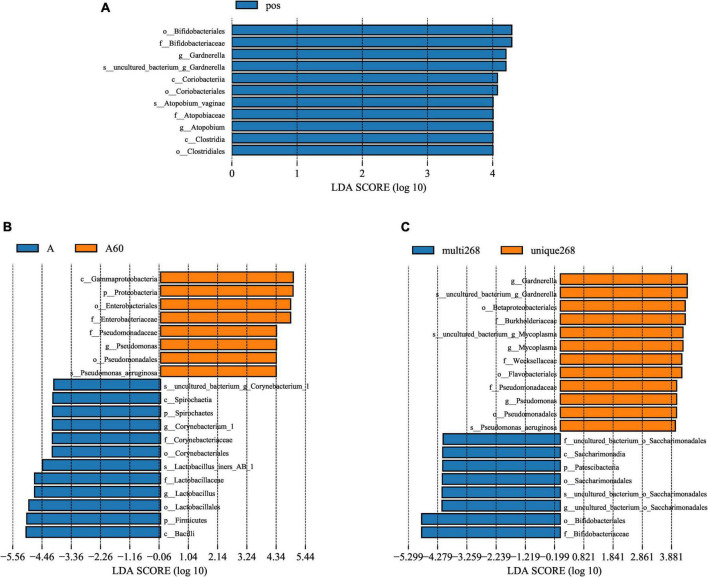
Microbiota significant difference analysed by LEfSe. **(A)** HPV-positive and HPV-negative patient groups, **(B)** HPV-positive single infection and multiple infection patient groups, and **(C)** age above and below 60 years old patient groups. LDA score threshold set as 4, above 4 will be shown in charts. LDA, linear discriminant analysis; LEfSe, LDA effective size. HPV, Human Papillomavirus. k_: kingdom, p_: phylum, c_: class_, o_: order, f_: family_, g_: genus.

Linear discriminant analysis (LDA) score was used to compare the different bacteria of each group. The results showed that *Bifidobacteriales* (order), *Bifidobacteriaceae* (family), *Gardnerella* (genus), *Coriobacteriia* (class), *Atopobium vaginae* (species), and *Clostridia* (class) were higher in HPV-infected group compared with the negative (Figure 2A). Between multi-268 and unique-268 groups, *Betaproteobacteriales* (order), *Burkholderiaceae* (family), *Weeksellaceae* (family), *Flavobacteriales* (family), *Gardnerella* (genus), *Pseudomonas aeruginosa* (species), and *Mycoplasma* (genus) were found with higher relative abundance in unique-268. In contrast, *Saccharimonadales* (order), *Saccharimonadia* (class), *Patescibacteria* (phylum), *Bifidobacteriales* (order), and *Bifidobacteriaceae* (family) were higher in the multi-268 group (Figure 2B). Among them, *Bifidobacteriaceae* was the most significantly different between the two groups, indicating its strong association with multi-268 infection (Figure 2B). *Corynebacterium* (genus), *Lactobacillus iners AB-1* (species), *Bacilli* (class), and *Firmicutes* (phylum) were identified higher in the group with age younger than 60 and *Gamma-proteobacteria* (class) and *Pseudomonas* (genus) higher in the group older than 60 years old (Figure 2C).

### Microbiota function difference of subjects group was identified

**FIGURE 3:**
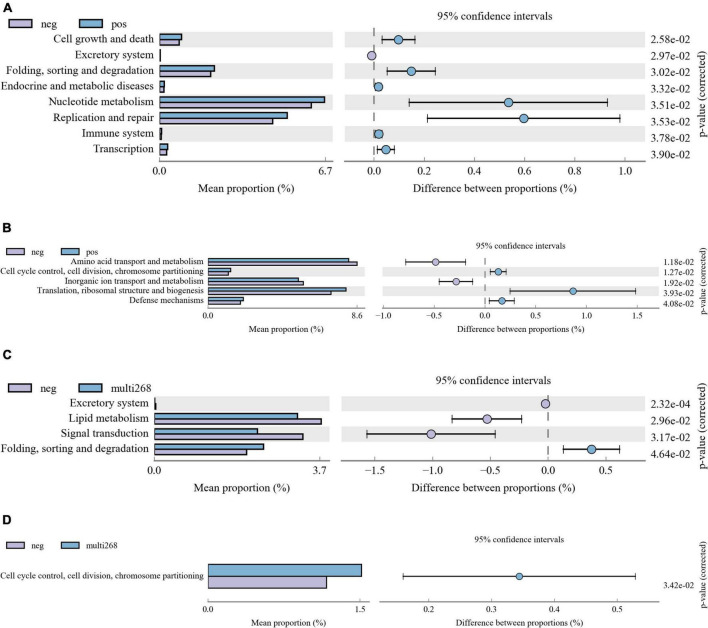
Microbiota KEGG and COG function difference diagrams. The picture shows the difference analysis diagram of the KEGG (and COG) metabolic pathway under the second level (also can be analysed for the third or first level): the different colours in the picture represent different groups. The figure shows the abundance ratio of different functions in the two sets of sample groups, the middle shows the difference ratio of the function abundance within the 95% confidence interval, and the rightmost value is the *p*-value. **(A)** KEGG pathway difference between HPV-positive and HPV-negative patient groups, **(B)** COG pathway difference between HPV-positive and HPV-negative patient groups. **(C)** KEGG pathway difference between multi-268 HPV infections and HPV-negative patient groups, **(D)** COG pathway difference between multi-268 HPV infections and HPV-negative patient groups. KEGG, Kyoto Encyclopedia of Genes and Genomes; COG, Clusters of Orthologous Groups.

Kyoto Encyclopedia of Genes and Genomes and COG analysis were applied, and functional difference between groups was explored. Among the three group comparisons, two group pairs were found significantly different and they are HPV-negative/positive group pair and the multi-268/negative group pair. No significant function difference was identified between Age groups. Between HPV-positive and negative groups, KEGG pathways, including Cell growth and death, Excretory system, Folding, sorting, and degradation, Endocrine and metabolic diseases, Nucleotide metabolism, Replication and repair, Immune system, and Transcription, were significantly different (Figure 3A). Moreover, the COG categories of Amino acid transport and metabolism, Cell cycle control, cell division, chromosome partitioning, Inorganic ion transport and metabolism, Translation, ribosomal structure and biogenesis and Defence mechanisms between the two groups were significantly different (Figure 3B). Comparison analysis results of multi-268 and the negative group showed that KEGG pathway Excretory system, Lipid metabolism, Signal transduction and Folding, sorting and degradation and COG categories of Cell cycle control, cell division, chromosome partitioning were significantly different (Figures 3C,D).

### Complexed cervical microbiota network relationships existed in the cervical microbiota system

**FIGURE 4:**
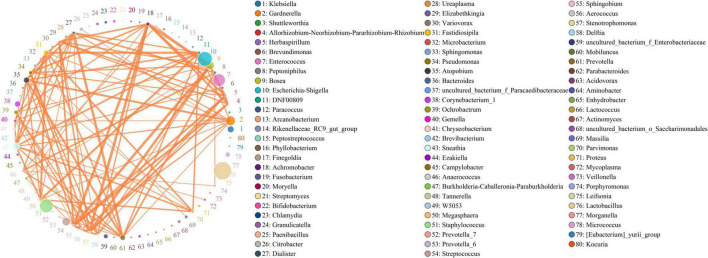
Microbiota network diagram. The top 50 most relevant genera were shown here. The circle in the figure represents the species, and the size of the circle represents the abundance; the line represents the relation between the two species, the thickness of the line represents the strength of the relation, and the colour of the line: orange represents positive correlation, and the green represents negative correlation.

Co-occurrent analysis of identified microbiota in cervical samples and results are shown in Figure 4. There were 80 genera of bacteria identified with more than seven relationships with other bacteria, and the correlation was higher than 10%, with a *p*-value less than 0.05. A network relationship was identified between them (Figure 4). The top 50 bacteria with high correlation were shown in the figure (Figure 4), 24 of which were correlated with two or more other genera. The abundance of the genera was different, ranging from 12.0 to 15463.4, and no significant correlation was observed between genera abundance and its correlation with other genera. The abundance of *Lactobacillus* (abundance of 15463.4), *Escherichia-Shigella* (10609.8), and *Staphylococcus* (9270.3) were high, but the correlation with other bacteria is relatively low, less than 0.34, 0.32, and 0.27, respectively. On the contrary, some genera’s abundance was relatively low, but the correlations with others were quite high (Supplementary Table 6). For example, *Atopobium* (abundance: 1510.4) is highly correlated to *Dialister* (correlation: 0.675, abundance: 236.2), *Prevotella* (0.656, 1259.3) and *Fastidiosipila* (0.573, 1121.7); *Achromobacter* (483.5) is tensely correlated to *Stenotrophomonas* (0.793, 305.4), *Sphingobium* (0.759, 241.9), and *Herbaspirillum* (0.648, 21.8); *Gardnerella (4349.4)* is highly correlated to *Atopobium* (0.659, 1510.4), *Aerococcus* (0.527, 246.2) and *Sneathia* (0.0.498, 2389.7); *Sneathia* (2389.7) is highly correlated to *Fastidiosipila* (0.648, 1121.7), DNF00809 (0.604, 242.4), *Parvimonas* (0.572, 278.8), and *Atopobium* (0.546, 1510.4). In summary, a complexed bacteria network relationship was existing in cervical system and the interactions between genera was not correlated with its abundance.

## Discussion

Data analysis showed that changes in the cervical microbiome, especially anaerobic bacteria, were significantly correlated with HPV infection status. *Gardnerella*, *Atopobium vaginae*, and *Sneathia* were the three most increased amongst microbiota, and these microorganisms form pathogenic biofilms through close cooperation. *Gardnerella* acts as a “scaffold” for biofilms (Harwich et al., 2010; Fethers et al., 2012; Curty et al., 2017; Khan et al., 2020), promoting the growth of *Atopobium vaginae* (Libby et al., 2008; Anahtar et al., 2015), *Sneathia* and other related pathogens by altering the microenvironment (Lee et al., 2013; Zhou et al., 2020). The growth of these pathogens has led to a rise in microbial diversity. Not only that, the cellular pro-inflammatory responses that these pathogenic microorganisms elicited will affect cellular metabolisms (Kacerovsky et al., 2015; Mitra et al., 2015, 2020; Onderdonk et al., 2016; Gosmann et al., 2017; Khan et al., 2020), such as amino acid transport (Mitra et al., 2020) and inorganic ion transport, and even cell shedding (Harwich et al., 2012; Africa et al., 2014). This deteriorates the immune response and leads to a defection of the microenvironment (Anahtar et al., 2015), making cervix HPV susceptible and possibly leading to the cervical cancer. Therefore, these three microorganisms are of great significance as biomarkers in the clinical identification of HPV infection.

In addition to the correlation with HPV, there is also a relationship between CM and age status. Lee et al. (2013) suggested that the decline in oestrogen and progesterone levels in the female reproductive tract after menopause is associated with an increased proportion of anaerobic bacteria. Several studies have also confirmed that older women have a higher proportion of anaerobic bacteria, and the biofilm produced by them is an important factor in HPV susceptibility (Singh et al., 2015; Zhou et al., 2020). Besides, Lee’s experiment also evidenced that due to the presence of oestrogen and progesterone, the proportion of *Lactobacillus* in young women is higher to maintain the homeostasis of the reproductive tract microenvironment (Lee et al., 2013; Oh et al., 2015). In contrast, the proportion of *Lactobacillus* in older women is lower, so it insufficiently maintains the homeostasis of the internal environment, making the proportion of γ-proteobacteria and Pseudomonas species increase as biomarkers.

However, this study also showed some results that differed from the prevailing view. In this study, the proportion of *Lactobacillus* in the HPV-positive group was higher than that in the normal group. It contradicts the mainstream ideas that the presence of *Lactobacillus* can maintain pH stability (Larsson et al., 1991; Brotman et al., 2014) and homeostasis in the reproductive tract (Mitra et al., 2016; Borgogna et al., 2020) and is therefore reduced in the HPV-positive group. However, a report from Iran showed the same results as this research and concluded that the proportion change of *Lactobacillus* was not strongly correlated with HPV infection status but did not rule out the influence of factors such as customs on sample interference (Ghaniabadi et al., 2020). Therefore, the influence of other factors, such as personal habits, could not be ruled out for the presence of interference with the sample microbiota. The more detailed mechanisms still need more experiments to verify and analyse.

Aside from that, this study also presented some new findings. A higher abundance of *Bifidobacterium* was also found in the multi-268 group than in the unique-268 group when comparing the two case samples. Under such circumstances, the same bacteria have different environmental adaptations in different case samples. Besides, by analysing the low-abundance flora of different groups, it was found that there were differences in the microbial composition of different HPV infection states. Comparing the LEfSe analysis of unique-268 and multi-268, it could be seen that some low-abundance bacterial groups play important roles in different HPV-infected samples. In the unique-268 group of patients, *Burkholderiaceae*, a pathogenic bacteria, could sensitise cells to HPV (Brenner et al., 2005). *Mycoplasma* could promote HPV penetration, survival and persistence, and it is frequently present in high-risk HPV patients (Biernat Sudolska et al., 2011; Ye et al., 2018; Wei et al., 2021). *Pseudomonas aeruginosa* is a prevalent factor in high-risk HPV samples, especially in the cancerous cervix (Werner et al., 2012; Di Paola et al., 2017; Zhang et al., 2021). The low-abundance species *Saccharimonadales*, *Saccharimonadia*, and *Patescibacteria* in the multi-268 group were all related to the synthesis of compound elements (Herrmann et al., 2019; Lemos et al., 2019; Tian et al., 2020; Hosokawa et al., 2021; Mason et al., 2021; Zhou et al., 2021; Wang et al., 2022). The results of the above microbiota under different HPV infection statuses have clinical implications for biomarkers for identifying cases.

In addition to the above microorganisms, this study also found that the microbiota (especially pathogenic microorganisms) significantly impacted metabolic function. Apart from the abnormal cellular metabolism mentioned above, differences in genetic metabolism, lipid metabolism, signal transduction and cell cycle metabolism were also detected between the HPV-positive group and the multi-268 group. Abnormalities in these functions are likely associated with increased microbial diversity and an increased proportion of pathogenic microorganisms (Mitra et al., 2020). However, there are few studies in this regard, so further experiments are needed to explore their relationship.

This study analysed the possible effects of cervical microbiome changes from different aspects. However, due to the limited number of statistical samples in the research process, we could not perform significant statistics for some more refined HPV genotypes. In addition, the lack of clinical information about patients (such as smoking, eating and other behaviours that may cause cervical cancer) also interfered with the experiment to a certain extent. However, the data analysis of this experiment still provides a sufficient factual basis and data support for clinical examination. Meanwhile, the microbial changes of single-infection and multi-infection case samples and the differences in metabolic functions under different HPV infection conditions were compared from a new perspective.

## Conclusion

Overall, the characteristics of cervical samples microbiota were explored in this study. *Escherichia coli, Enterococcus faecalis*, and *Atopobium vaginae* in species level, *Staphylococcus, Lactobacillus* (excluding *Lactobacillus iners AB1*), *Gardnerella, Bosea, Streptococcus*, and *Sneathia* in genus level were found as high abundant bacteria in studied samples. Microbiota composition was related to HPV infection status and age, which further influenced the diversity. Specific bacteria were identified with significantly different abundance between groups. For instance, compared with unique-268, *Bifidobacteriaceae* impacted more on the multi-268 group. Moreover, some low abundance bacteria also play a vital role in specific HPV infections, such as *Saccharimonadales*, *Saccharimonadia*, and *Patescibacteria* in multi-268, *Burkholderiaceae Mycoplasma*, and *Pseudomonas aeruginosa* in unique-268. Besides, the different composition of microbiota also affected the disparities of function pathways to the metabolism of the cell, protein and genetic information between HPV infection and HPV-negative groups, and the metabolism of lipid, signal transduction and cell cycle between multi-268 infection and HPV-negative groups. In summary, our study descriptively explored the microbiota characteristics of cervical samples from Sanmenxia area patients. The analysis of single infections was not developed due to the sample size. The research concerns specific single infections, and CIN could be further investigated into their microbiota in future works.

## Data availability statement

The datasets presented in this study can be found in online repositories. The names of the repository/repositories and accession number(s) can be found below: https://www.ncbi.nlm.nih.gov/, PRJNA795603.

## Ethics statement

The studies involving human participants were reviewed and approved by the Ethics Committee, Sanmenxia Central Hospital. Written informed consent for participation was not required for this study in accordance with the national legislation and the institutional requirements.

## Author contributions

JH contributed to the manuscript writing, revision, and partial statistical data analysis. YW contributed to the initial draft discussing, editing and the general ideas of the manuscript, and collection of literature. LQ contributed to all the sample collection, ethic letter application, and results discussion. JL contributed to the part of the data analysis and data subscribing. WY did the experiments of 16S rDNA sequencing. GT contributed to the conceptualization of the study, study fund support, and results discussion. BM contributed to the conceptualization of the study, data generation and analysis, and draft revision. All authors read and revised the manuscript.

## Footnotes

1
http://www.Bioinformatics.babraham.ac.uk/projects/fastqc/

2
http://hannonlab.cshl.edu/fastx_toolkit/
