# Supplementary material for: Research of cervical microbiota alterations with human papillomavirus infection status and women age in Sanmenxia area of China
Source: Front Microbiol. 2022 Oct 6;13:1004664. doi: 10.3389/fmicb.2022.1004664 (PMC9608786; doi:10.3389/fmicb.2022.1004664)
Supplement: Supplementary file 1 [file Presentation_1.PPTX]

## Slide 1
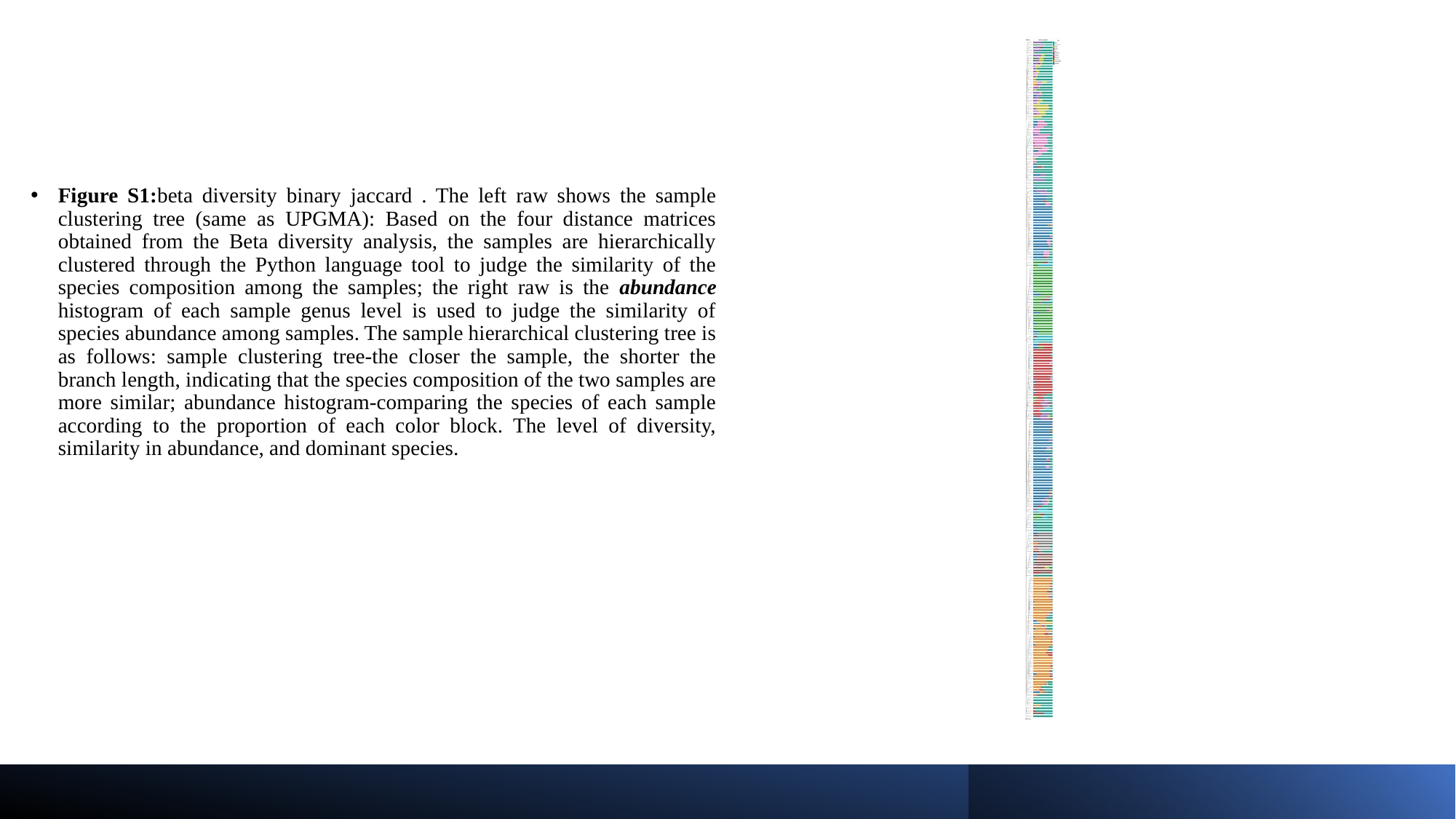

Figure S1:beta diversity binary jaccard . The left raw shows the sample clustering tree (same as UPGMA): Based on the four distance matrices obtained from the Beta diversity analysis, the samples are hierarchically clustered through the Python language tool to judge the similarity of the species composition among the samples; the right raw is the abundance histogram of each sample genus level is used to judge the similarity of species abundance among samples. The sample hierarchical clustering tree is as follows: sample clustering tree-the closer the sample, the shorter the branch length, indicating that the species composition of the two samples are more similar; abundance histogram-comparing the species of each sample according to the proportion of each color block. The level of diversity, similarity in abundance, and dominant species.
